# Metformin Suppressed CXCL8 Expression and Cell Migration in HEK293/TLR4 Cell Line

**DOI:** 10.1155/2017/6589423

**Published:** 2017-09-24

**Authors:** Zhihui Xiao, Wenjun Wu, Vladimir Poltoratsky

**Affiliations:** Department of Pharmaceutical Sciences, College of Pharmacy and Health Sciences, St. Johns University, 8000 Utopia Parkway, Jamaica, NY 11439, USA

## Abstract

Chronic inflammation is associated with cancer. CXCL8 promotes tumor microenvironment construction through recruiting leukocytes and endothelial progenitor cells that are involved in angiogenesis. It also enhances tumor cell proliferation and migration. Metformin, type II diabetes medication, demonstrates anticancer properties via suppressing inflammation, tumor cell proliferation, angiogenesis, and metastasis. This study intended to address the role of metformin in regulation of CXCL8 expression and cell proliferation and migration. Our data indicated that metformin suppressed LPS-induced CXCL8 expression in a dose-dependent manner through inhibiting NF-*κ*B, but not AP-1 and C/EBP, activities under the conditions we used. This inhibitory effect of metformin is achieved through dampening LPS-induced NF-*κ*B nuclear translocation. Cell migration was inhibited by metformin under high dose (10 mM), but not cell proliferation.

## 1. Introduction

Chronic inflammation is associated with high incidence of various types of cancer, including cervical, gastric, and intestinal cancers [1]. In response to inflammatory stimuli, leucocytes release reactive oxygen and nitrogen species that cause DNA lesions, including oxidized bases, single and double DNA breaks, which lead to genome instability. Probability of tumor formation increases with the accumulation of mutations in oncogenes and/or tumor suppressor genes [2–4].

Malignant cells created inflammatory microenvironment by releasing inflammatory cytokines and chemokines, in particular interleukin- (IL-) 8 (CXCL8). CXCL8 recruits leukocytes, such as monocytes and neutrophils, into tumor microenvironment. Infiltrated macrophages and neutrophils, in their turn, release growth factors and proteases that promote angiogenesis and tumor metastasis. The level of tumor-associated macrophage and neutrophil infiltration closely correlates with poor prognosis in breast, prostate, lung, and melanoma cancers [5–9].

CXCL8 is a member of inflammatory chemokine family that promotes chemotaxis by activating CXCR1 or CXCR2 receptors on targeted cells [10–12]. These receptors are expressed in neutrophils, monocytes, mast cells, eosinophils, natural killer (NK) cells, and activated CD8^+^ T cells [13–16]. CXCL8 mediates recruitment of these immune cells and endothelial progenitor cells, regulating inflammation, angiogenesis, and wound healing. In cancer, CXCL8 promotes tumor cell proliferation and migration, angiogenesis, and metastasis [17–21].

CXCL8 is overexpressed in multiple cancer types, including nonsmall cell lung cancer (NSCLC) [22, 23], breast [24, 25], pancreatic [26], and colorectal cancers [21]. In clinical studies, patients with high CXCL8 levels are reported to have poorer prognosis: lower survival rate, higher liability of tumor recurrence after surgery excision, and higher liability of tumor metastasis to distant organs [26]. Therefore, CXCL8 can be used as a predictor for tumor prognosis, at least for pancreatic [26] and colorectal cancers [27].

CXCL8 expression is regulated primarily by three transcription factors: nuclear factor kappa-light-chain-enhancer of activated B cells (NF-*κ*B), activator protein 1 (AP-1), and CCAAT/enhancer binding protein (C/EBP). In malignant cells, CXCL8 expression is mediated through NF-*κ*B [20]. Transcription factor NF-*κ*B is originally synthesized as a protein complex containing the NF-*κ*B and the inhibitor of *κ*B (I*κ*B). In response to stimuli, I*κ*B is phosphorylated by I*κ*B kinase (IKK) and then undergoes degradation resulting in release of NF-*κ*B. The released NF-*κ*B, which consists of p50 and p65 subunits, translocates into nucleus and binds to the binding site in CXCL8 promoter, triggering gene expression.

Metformin is the first-line drug for type II diabetes (T2D) [28]. It is a biguanide drug that modifies the glucose metabolism by activating the adenosine monophosphate-activated protein kinase (AMPK) signaling pathway. AMPK activation inhibits gluconeogenesis in the liver [29, 30] and absorption of glucose in the intestine [31, 32].

Diabetic patients are at a higher risk for cancer due to chronic inflammatory conditions [33]. However, meta-analysis revealed that T2D patients who are treated with metformin demonstrated lower incidence of cancer [34]. Metformin is associated with suppression of the inflammatory responses, thereby reducing the liability of tumor formation. It also suppresses proliferation of tumor cells in various cancers [35, 36]. In our research, we focused on the potential role of metformin in CXCL8 expression, the key factor that orchestrates tumor microenvironment formation.

## 2. Materials and Methods

### 2.1. Cell Culture

The HEK293/TLR4 cells expressing TLR4, MD2, and CD14 (InvivoGen, CA) were cultured in Dulbecco's modified Eagle's media (DMEM, Hyclone, UT) containing 10% fetal bovine serum (FBS, ATLANTA biologicals, GA) and 1% antibiotic/antimycotic solution (Hyclone, UT), at 37°C and 5% CO_2_.

### 2.2. Cell Viability Assay

HEK293/TLR4 cells were plated into 96-well plates. Next day, the cells were cultured with different concentrations of metformin for 24 h. After treatment, cells were treated with 3-(4,5-dimethylthiazol-2-yl)-5-(3-carboxymethoxyphenyl)-2-(4-sulfophenyl)-2H-tetrazolium (MTT, Acros, NY) for additional 2 h. The supernatant was aspirated, and the formazan crystal was dissolved in dimethylsulfoxide (DMSO, Thermo Scientific, MA). The absorbance intensity was measured by using BioTek Synergy H1 Multi-Mode Reader (Biotek, VT) at 560 nm with a reference wavelength of 670 nm. The relative cell viability (%) was expressed as percentage relative to the untreated control cells.

### 2.3. ELISA Assay

HEK293/TLR4 cells were plated into 96-well plates. Next day, cells were pretreated with different concentrations of metformin (MP Biomedicals, CA) for 24 or 48 h and then incubated with or without 1 *μ*g/ml lipopolysaccharide (LPS, Sigma-Aldrich, MO) for additional 24 h. The concentration of secreted CXCL8 in the cell culture media was determined using ELISA assay.

The high binding half-area 96-well plates (Corning, NY) were coated with 1 *μ*g/ml antihuman IL-8 coating antibody (Invitrogen, MD) overnight. The plates were then washed with washing buffer (1.47 mM KH_2_PO4 and 8.32 mM K_2_HPO4, 0.05% Tween 20, pH 7.4) and blocked with assay buffer (13.69 mM NaCl, 7.69 mM Na_2_HPO4, 1.15 mM K_2_HPO4, and 2.68 mM KCl, 0.5% bovine serum albumin, 0.05% Tween 20, pH 7.4).

The cell culture media samples, IL-8 standards and antihuman IL-8 antibodies conjugated with biotin, were added to the ELISA plates and incubated for 2 h. Wells were washed and incubated with streptavidin-HRP solution for 30 min. The plates were then washed, and 1-Step™ Ultra TMB-ELISA (Thermo Scientific, MA) substrate was added to the plates and incubated in dark for 30 min. The HRP reaction was stopped by sulfuric acid, and absorbance was measured at 450 nm and 650 nm. CXCL8 concentration in samples was calculated and managed via Prism software (GraphPad Software, CA).

### 2.4. Luciferase Assay

NF-*κ*B, AP-1, or C/EBP plasmids were diluted in Opti-MEM media (Life Technologies, CA), and TransIT-LT1 reagent (Mirus, WI) was added to the diluted plasmids. The plasmid/reagent mixture was then added to HEK293/TLR4 cells and plated into 96-well plates. The transfected cells were treated with different concentrations of metformin for 24 h. Then, LPS was added to every well for another 24 h. The intracellular luciferase activity was determined using Pierce® Firefly Luciferase Glow Assay Kit (Thermo Scientific) according to manufacturer's instruction.

### 2.5. Immunofluorescence Assay

HEK293/TLR4 cells were cultured on poly-D-lysine-coated coverslides (GG-12-PPL, Neuvitro, WA) in 24-well plates. Next day, the cells were incubated with 0 or 10 mM metformin for 2 h, followed by a treatment with or without 1 *μ*g/ml LPS treatment for additional 15 min.

After incubation, cells were fixed with 4% paraformaldehyde (Sigma-Aldrich, MO), permeabilized with 0.1% TRITON-X (Sigma-Aldrich, MO), and blocked with 1% BSA. Cells were incubated with primary rabbit anti-NF-*κ*B-p65 (1 : 200, Santa Cruz, TX) at 4°C overnight and secondary Alexor 594 goat anti-rabbit (1 : 200, Life Technologies, CA) for 2 h. Cell nuclei were counter-stained with DAPI. Immunofluorescence analysis was performed using the Nikon Eclipse TE2000-S microscope (Nikon Instruments Inc., NY).

### 2.6. Wound-Healing Assay

The HEK293/TLR4 cells were plated into 6-well plates. The cells were incubated with 0, 0.1, 1, or 10 mM metformin for 24 h and then incubated with or without 1 *μ*g/ml LPS for another 24 h. A strait scratch was performed with pipette tips when confluent monolayers were formed. Cells were cultured for another 24 h. Three randomly selected stretched regions were pictured using a microscope (Nikon Eclipse TE2000-S, Nikon Instruments Inc., NY). Ten regions from each image were randomly chosen for distance measuring.

### 2.7. Cell Cycle (Flow Cytometry)

The HEK293/TLR4 cells were plated into 10 cm tissue culture dishes. The cells were incubated with different concentrations of metformin, followed by a treatment with or without LPS for additional 24 h. Cells were then harvested, washed with PBS (136.9 mM NaCl, 10.14 mM Na_2_HPO_4_, 1.38 mM K_2_HPO_4_, and 2.68 mM KCl, pH 7.4), and fixed with ice-cold 70% ethanol. The cells were then washed with PBS and PI/RNase Staining Buffer (BD Biosciences, CA). The cells were subsequently incubated with 0.5 ml PI/RNase Staining Buffer for 15 minutes. Cell cycle distribution was assessed by using BD Accuri C6 Flow Cytometer (BD Biosciences, CA). The statistical data are analyzed via BD Accuri C6 Software (BD Biosciences, CA).

### 2.8. Statistical Analysis

All experiments were done in triplicates and repeated for three times. Data from MTT, ELISA, luciferase, wound-healing assays, and cell cycle were presented as mean ± SEM. The data were analyzed by using one-way analysis of variance (ANOVA) followed by Newman–Keuls post hoc test for multiple comparisons. Significant difference was defined as *P* values less than 0.05.

## 3. Results

Chronic inflammation is associated with cancer and CXCL8 [1], one of the proinflammatory chemokines, promotes tumorigenesis, angiogenesis, and metastasis [19, 21, 24, 37, 38]. Clinical studies indicate that high plasma level of CXCL8 observed in cancer patients is associated with poorer prognosis [26]. It has been shown that metformin exhibits anti-inflammatory responses. We studied the effects of metformin on expression of CXCL8.

### 3.1. Metformin Cytotoxicity

**Figure 1:**
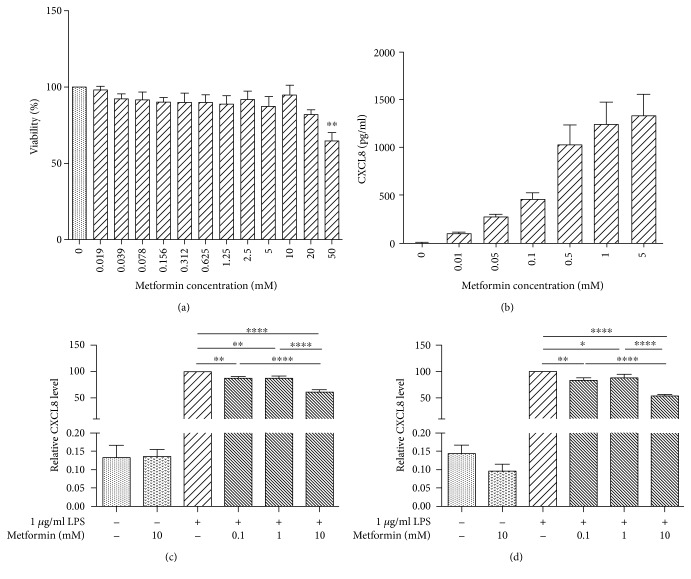
Metformin inhibited LPS-induced CXCL8 expression. (a) Metformin cytotoxicity. HEK294/TLR4/TLR4 cells were treated with serial concentrations of metformin for 24 h. Cytotoxicity was measured through using MTT assay. Data are reported as mean ± SEM. Statistically significant differences are indicated by an asterisk (^∗^*P* < 0.05, compared to control). (b) LPS-induced CXCL8 expression. HEK294/TLR4 cells were treated with serial LPS concentrations for 24 hours. CXCL8 concentration in the culture media was then measured by ELISA kit. Data are reported as mean ± SEM. (c, d) LPS-induced CXCL8 expression was suppressed by metformin pretreatment for 24 h (c) or 48 h (d). HEK294/TLR4 cells were treated with different concentrations of metformin for 24 or 48 h, followed by 24 h incubation with LPS. CXCL8 concentration in the culture media was measured via using ELISA kit. Data are normalized by CXCL8 concentration of cells treated with LPS only. ^∗^*P* < 0.05, ^∗∗^*P* < 0.01, and ^∗∗∗∗^*P* < 0.0001 compared to 1 *μ*g/ml LPS group.

Cytotoxicity of metformin was determined using MTT assay (Figure 1(a)). We observed no toxic effect of metformin at doses from 0 to 20 mM. Metformin causes significant toxicity under 50 mM (64.4 ± 5.654%, *P* < 0.01). From the cytotoxic study of metformin, nontoxic doses of 0.1, 1, and 10 mM were selected for use in our following experiments.

### 3.2. LPS-Induced CXCL8 Production Is Suppressed by Metformin

In the malignant cells, regulation of CXCL8 production is associated with complicated interactions of multiple signaling pathways. To simplify the regulatory system that modifies CXCL8 expression, we employed HEK293 cells expressing TLR4, MD2, and CD14. In HEK293/TLR4 cells, CXCL8 expression was induced by TLR4 ligand, LPS.

We first examined the inductive effect of LPS on CXCL8 expression (Figure 1(b)). The CXCL8 levels of the cells treated with 0, 0.01, and 0.05 *μ*g/ml LPS were 1.294 ± 0.5504, 95.79 ± 18.25, and 269.2 ± 34.75 pg/ml, respectively. CXCL8 concentration of the cells treated with 0.5 *μ*g/ml LPS (1028 ± 213.5 pg/ml) was proximately twofolds higher than that of the cells treated with 0.1 *μ*g/ml LPS (452.5 ± 74.03 pg/ml). Cells treated with 0.5, 1, and 5 *μ*g/ml LPS exhibited no differences in CXCL8 levels (1028 ± 213.5, 1240 ± 239.3, 1332 ± 229.9 pg/ml, resp.), indicating saturation on LPS-induced CXCL8 expression. Overall, LPS induce CXCL8 expression in a dose-dependent manner in HEK293/TLR4 cells. Based on this data, 1 *μ*g/ml LPS would be used in the subsequent experiments.

We then studied the effect of metformin on LPS-induced CXCL8 expression under nontoxic doses of 0.1, 1, and 10 mM. The cells were pretreated with metformin for 24 h (Figure 1(c)) or 48 h (Figure 1(d)), followed by LPS treatment for 24 h. The relative CXCL8 levels were expressed as percentage to the CXCL8 concentration of the cells treated with 1 *μ*g/ml LPS (only).

Significant differences on the relative CXCL8 levels were observed in the cells pretreated with 0.1 mM (87.76 ± 2.946%, *P* < 0.01), 1 mM (86.95 ± 4.806%, *P* < 0.01), and 10 mM (61.14 ± 4.508%, *P* < 0.0001) metformin, as compared to those in the cells treated with 1 *μ*g/ml LPS (only). Significant differences on relative CXCL8 levels were also observed between the cells pretreated with 10 mM (*P* < 0.0001) metformin and the cells pretreated with 0.1 and 1 mM metformin, indicating a dose-dependent inhibitory effect of metformin. However, no statistical difference on relative CXCL8 levels was detected between the LPS-induced cells pretreated with 0.1 and 1 mM metformin.

Similar inhibitory effect of metformin on CXCL8 expression was also observed in the cells pretreated with metformin for 48 h. In the LPS-stimulated cells, the relative CXCL8 levels of the cells pretreated with 0, 0.1, 1, and 10 mM metformin for 48 h were 100%, 82.60 ± 5.428% (*P* < 0.01), 88.09 ± 7.083% (*P* < 0.05), and 53.67 ± 2.966% (*P* < 0.0001), respectively, indicating the suppressive effect of metformin on CXCL8 production. Statistical analysis exhibited significant differences between the low-dose (0.1 and 1 mM) and high-dose (10 mM) metformin-pretreated cells (*P* < 0.0001). No difference on the relative CXCL8 levels was observed between the 0.1 and 1 mM metformin-pretreated cells.

There was no difference in CXCL8 levels between the corresponding 24 h and 48 h metformin pretreatment groups (data not shown), indicating that the inhibitory effect of metformin on CXLC8 production was not in time-dependent fashion.

### 3.3. LPS-Induced CXCL8 Production Is Mediated through Transcriptional Factor NF-*κ*B

Three transcriptional factors play the major role in transcriptional regulation of CXCL8 expression: AP-1, C/EBP, and NF-*κ*B [12]. We investigated the role of these transcription factors in LPS-induced CXCL8 expression using luciferase assay.

**Figure 2:**
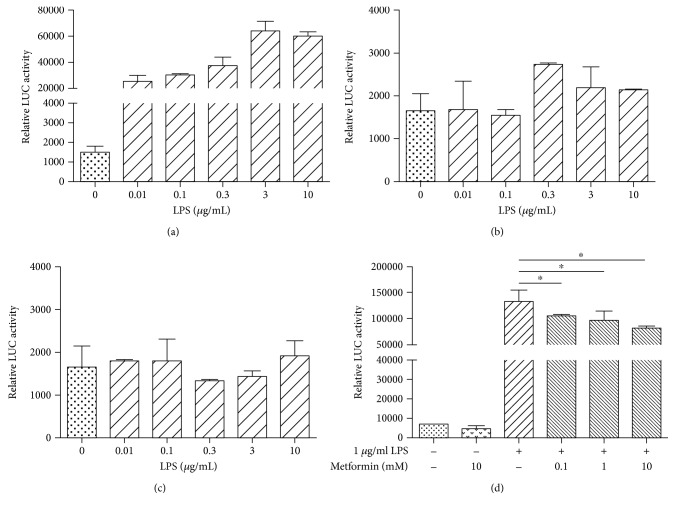
Metformin suppressed LPS-induced NF-*κ*B activities. HEK294/TLR4 cells were transfected with plasmids that contain transcription factors NF-*κ*B, AP-1, and C/EBP binding sites upstream the luciferase sequence. The transfected cells were treated with different concentrations of metformin and LPS. Luciferase activity was assessed through luciferase assay. (a) NF-*κ*B relative luciferase activity induced by LPS. (b) AP-1 relative luciferase activity induced by LPS. (c) C/EBP relative luciferase activity induced by LPS. (d) Metformin (24 h treatment) inhibited LPS-induced NF-*κ*B relative luciferase activity. ^∗^*P* < 0.05.

In the NF-*κ*B plasmid-transfected cells (Figure 2(a)), the relative luciferase activities of the 0, 0.01, 0.1, 0.3, 3, and 10 *μ*g/ml LPS-treated cells were 1502 ± 313.5, 12,600 ± 81.5, 18,469 ± 417, 27,424 ± 7810, 60,255 ± 8952, and 55,745 ± 3565, respectively. Significant differences were observed between the 0 *μ*g/ml LPS-treated cells and the cells treated with 0.3 *μ*g/ml (*P* < 0.05), 3 *μ*g/ml (*P* < 0.01), and 10 *μ*g/ml (*P* < 0.01), indicating the involvement of the transcription factor NF-*κ*B in LPS-induced CXCL8 gene transcription. There was no significant difference on relative luciferase activity between the 3 *μ*g/ml and 10 *μ*g/ml LPS-treated cells, indicating a saturation of the inductive effect of LPS. In contrast, we did not observe LPS-induced luciferase activities in the cells transfected with AP-1 or C/EBP plasmids (Figures 2(b) and 2(c)) suggesting that AP-1 and C/EBP were not involved in LPS-induced transcriptional modification in HEK293/TLR4 cells under the conditions we used.

We then studied the effect of metformin on the NF-*κ*B activities using luciferase assay (Figure 2(d)). HEK293/TLR4 cells were transfected with NF-*κ*B plasmid. In the LPS-stimulated cells, the relative luciferase activities of the cells pretreated with 0, 0.1, 1, and 10 mM metformin were 133,169 ± 14,721, 104,680 ± 3528, 96,359 ± 12,658, and 81,388 ± 3322, respectively. Significant differences were observed in the cells pretreated with 0.1, 1, and 10 mM of metformin (*P* < 0.05) as compared to the cells treated with LPS (only), suggesting the suppressive effect of metformin on NF-*κ*B activities.

### 3.4. Metformin Inhibited LPS-Induced Nuclear Translocation of NF-*κ*B

**Figure 3:**
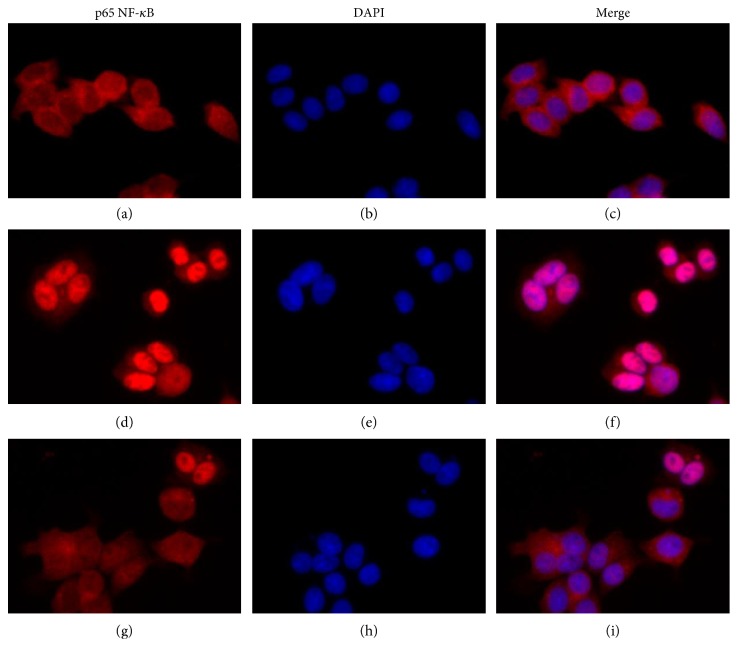
Metformin inhibited LPS-induced NF-*κ*B translocation. HEK294/TLR4 cells were treated with or without metformin for 2 h, followed by 15 min induction with 1 *μ*g/ml LPS. Untreated cells were used as a control. After fixation and permealization steps, cells were stained with antibody against subunit p65 NF-*κ*B and counterstained with DAPI. (a, b, c) Cells without treatment; (d, e, f) cells treated with 1 *μ*g/ml LPS; (g, h, i) cells treated with 10 mM metformin and 1 *μ*g/ml LPS. (a, d, g) anti-p65 antibody; (b, e, h) DAPI counterstaining; (c, f, g) merged image p65/DAPI.

As it was shown by the luciferase assay, NF-*κ*B was involved in regulation of the LPS-induced CXCL8 expression in HEK293/TLR4 cells. We further studied the effect of metformin on LPS-induced NF-*κ*B translocation (Figure 3) using immunofluorescence assay. NF-*κ*B complexes were detected by using antihuman p65 antibody (Figures 3(a), 3(d), and 3(g)). Nucleuses of the cells were counter-stained with DAPI (Figures 3(b), 3(e), and 3(h)). The merged images were shown in Figures 3(c), 3(f), and 3(i).

Under the unstimulated state, NF-*κ*B was trapped in cytoplasm (Figure 3(c)). NF-*κ*B nuclear translocation was observed in the cells stimulated with LPS (Figure 3(f)). Metformin suppressed this LPS-induced NF-*κ*B nuclear translocation (Figure 3(i)), indicating that metformin suppressed NF-*κ*B activities, at least partially, through inhibiting nuclear translocation.

### 3.5. Wound-Healing Model: Proliferation and Migration

**Figure 4:**
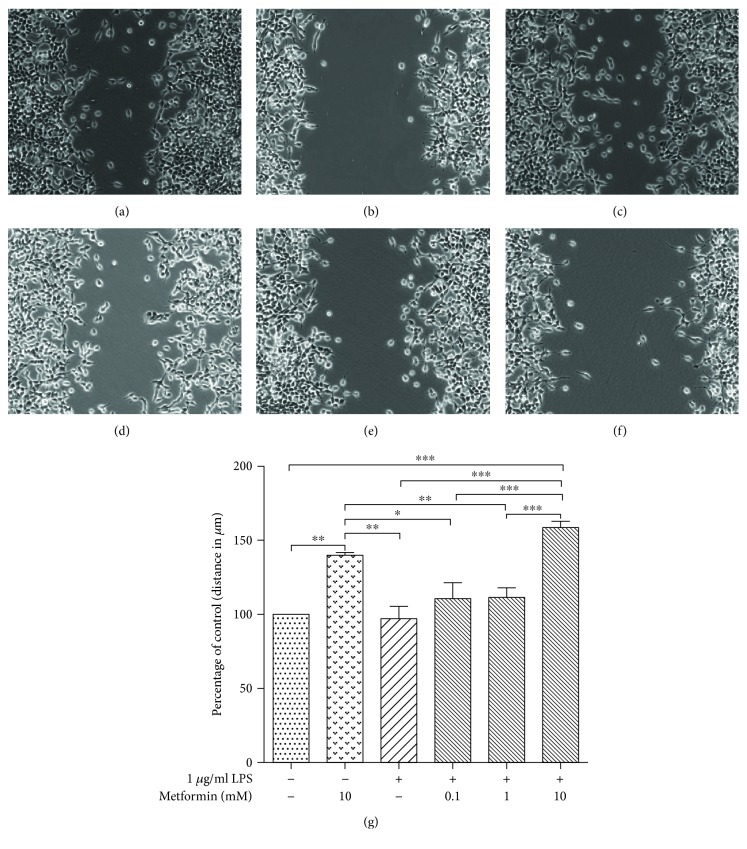
Metformin suppressed HEK294/TLR4/TLR4 cell migration. HEK294/TLR4 cells were treated with different concentrations of metformin for 24 h, followed by 1 *μ*g/ml LPS treatment for 24 h. A scratch was then made when cells formed monolayer. Images were taken after another 24-hour culture. (a) Control, (b) 10 mM metformin, (c) 1 *μ*g/ml LPS, (d) 0.1 mM metformin and 1 *μ*g/ml LPS, (e) 1 mM metformin and 1 *μ*g/ml LPS, and (f) 10 mM metformin and 1 *μ*g/ml LPS cells. (g) Plot of relative migration distances (expressed as the distance between the boarders of the scratch to that of the cells treated with LPS only). ^∗^*P* < 0.05, ^∗∗^*P* < 0.01, and ^∗∗∗^*P* < 0.001.

Evidence indicated that metformin suppresses tumor cell proliferation and migration [39–41]. We used a wound-healing model to investigate the effect of metformin on HEK293/TLR4 cell proliferation and migration (Figure 4). The images of the wound-healing model were shown in Figures 4(a), 4(b), 4(c), 4(d), 4(e), and 4(f). Ten distances between the boards of the scratch were measured. The relative distances were expressed as percentage to the distances of the untreated cells (Figure 4(g)).

The relative distances of the LPS-stimulated cells pretreated with 0, 0.1, and 1 mM metformin were 97.29 ± 8.205%, 110.6 ± 10.75%, and 111.3 ± 6.779%, respectively. No significant difference was observed among those groups of cells. The relative distance of the LPS-stimulated cells pretreated with 10 mM metformin was 158.7 ± 4.323%, exhibiting a significant difference compared to that of the cells treated with LPS only (*P* < 0.001). Interestingly, the cells treated with 10 mM metformin (only) had a relative distance of 139.9 ± 1.087%, which was significantly different with the cells treated with LPS only (*P* < 0.05). Accordingly, we concluded that high dose of metformin (10 mM) suppressed either proliferation or migration, or both proliferation and migration of the HEK293/TLR4 cells. However, this inhibitory effect of metformin on the cell proliferation and migration may be independent from the suppressive effect on the transcription factor NF-*κ*B, which required further investigation.

**Figure 5:**
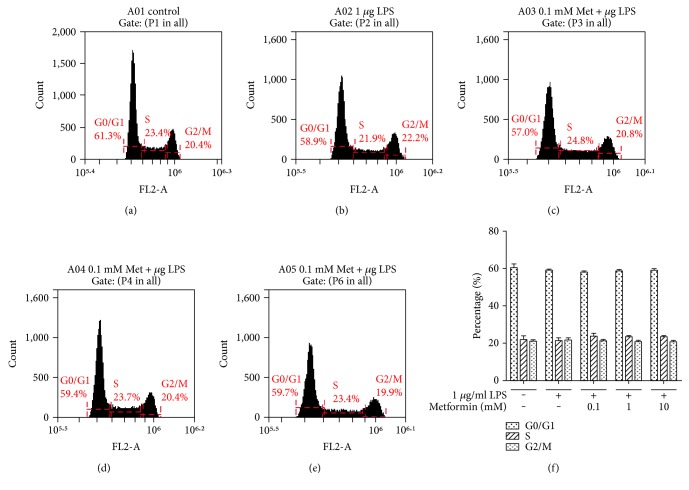
Metformin effects on cell cycle. HEK294/TLR4 cells were treated with different concentrations of metformin for 24 hours, followed by incubation with 1 *μ*g/ml LPS for 24 hours. The cells were then washed and fixed. Nucleus was stained with PI. Cell cycle was assessed by using flow cytometer. Representative results in (a) control, (b) 1 *μ*g/ml LPS, (c) 0.1 mM metformin and 1 *μ*g/ml LPS, (d) 1 mM metformin and 1 *μ*g/ml LPS, and (e) 10 mM metformin- and 1 *μ*g/ml LPS-treated cells. (f) Histogram of cell cycle data. Data are reported as mean ± SEM.

To elucidate the effect of metformin on HEK293/TLR4 cell proliferation, we examined cell cycle progression under nontoxic doses (Figure 5). According to our data, metformin did not affect HEK293/TLR4 cell cycle under the conditions we used. The MTT cell viability and proliferation study also revealed that metformin exerted no effect on cell proliferation (Figure 1(a)).

We concluded that metformin suppressed migration, but not proliferation, thereafter resulting in increase in distance between the scratch in the wound-healing model.

## 4. Discussion

CXCL8 is overexpressed in various cancer types [21–26]. CXCL8 has been considered to be a biomarker for various cancer types [42], and high serum level of CXCL8 levels in cancer patients is associated with poor prognosis [23, 26]. *In vivo* studies revealed that suppression of CXCL8 expression leads to tumor regression [43].

Recently, pharmaceutical agents that have the potential to suppress CXCL8 expression are being investigated for the potential use in cancer treatment. Metformin, the first-line medication for type II diabetes, exerts anti-inflammatory potentials [44–46]. It inhibits the expression of proinflammatory mediators, such as IL-1*α*, IL-1*β*, and IL-6, through suppressing NF-*κ*B activities. Evidence also shows that metformin suppresses tumor cell proliferation [46–48], as well as cell invasion and migration [39–41]. In our study, we investigate the potential role of metformin in inhibiting CXCL8 expression, as well as HEK293/TLR4 cell proliferation and migration.

Expression of CXCL8 is regulated at multiple levels, including chromatin modifications, transcription, mRNA processing, RNA stability, RNA interference, and posttranslational control. At a transcriptional level, CXCL8 expression is predominantly regulated by transcription factors NF-*κ*B, AP-1, and C/EBP. In this study, we demonstrated that LPS-dependent CXCL8 gene expression is triggered by activation of NF-*κ*B. LPS-induced activation of AP-1 and C/EBP was negligible under the conditions we used, and therefore we could not test the metformin effect on the activity of these transcription factors in HEK293/TLR4 cell line. Similar to what was observed by others, our results demonstrated that CXCL8 expression was regulated predominantly through LPS-dependent activation of NF-*κ*B pathway [49]. However, other studies indicated that CXCL8 expression also requires the conjoint action of multiple transcription factors. In Jurkat lymphoma and human gastric cancer cell line, activation of CXCL8 is mediated through cooperative action of AP-1 and NF-*κ*B [50, 51]. These alterations can be explained by differences in expression patterns of transcription factors and mediators of signal transduction pathways in different cell lines.

The NF-*κ*B is a key player in regulation of inflammatory responses [46]. Metformin suppressed LPS-induced CXCL8 expression predominantly through downregulating NF-*κ*B activity in our cell model. We observed significantly diminished LPS-induced NF-*κ*B p50-p65 dimer translocation in the cells pretreated with 10 mM metformin. This inhibitory effect of metformin was possibly achieved through activating AMPK signaling [40, 46, 52]. However, NF-*κ*B is not a direct substrate to AMPK. AMPK activates negative regulator of NF-*κ*B pathway, the NAD^+^-dependent protein deacetylase SIRT1. SIRT1 regulates NF-*κ*B activities by deacetylation of NF-*κ*B-p65 subunit, therefore inhibiting NF-*κ*B activities and promoting resolution of inflammation [53–55]. SIRT1 also modulates activities of other factors, including peroxisome proliferator-activated receptor gamma coactivator-1*α* (PGC-1*α*), p53, and Forkhead box O (FoxO) transcription factor [53–56]. To further understand metformin-mediated regulation on NF-*κ*B activities in our HEK293/TLR4 model, the activities of AMPK and its associated downstream targets require an additional study.

The anticancer properties of metformin could be partially explained by its effect on proliferation of tumor cells [47, 48, 57]. *In vivo* study showed that metformin reduced tumor volume and weight [46]. In contrast, in our study, metformin did not affect the proliferation of HEK293/TLR4 cells. This contrast could be explained partially by the differences in the cell lines. HEK293/TLR4 used in this study was normal human embryonic kidney cells, whereas malignant tumor cells, used by other groups, demonstrated sustained proliferation and high metabolic rate. Metformin may affect cells with high proliferative and metabolic rate, but not the normal tissue cells.

In our wound-healing model, metformin significantly inhibited the migration of HEK293/TLR4 cells under the high dose (10 mM). The migration of cells was independent on LPS treatment. Migration of the cells pretreated with 10 mM metformin was suppressed to the same level to that of the LPS-induced cells treated with 10 mM metformin. This result suggests that the effect of metformin on cell migration was independent from its inhibitory effect on NF-*κ*B activities. However, the correlation of cell migration and NF-*κ*B activities requires further study.

The HEK293/TLR4 cell line we used does not express CXCL8 receptors: CXCR1 and CXCR2. In the CXCR-positive tumor cells, the secreted CXCL8 acted in an autocrine manner promoting tumor cell proliferation, migration, and angiogenesis [21]. CXCL8 in CXCR-positive cells through activating CXCR1 and CXCR2 signals transduction pathways [21]. Activation of CXCR1 and CXCR2 signal transduction pathways enhances NF-*κ*B activities that further promote CXCL8 expression, generating a self-perpetuating cycle. In our study, the autocrine action of CXCL8 was excluded. Therefore, we were able to study CXCL8 expression induced by LPS solely and the effect of metformin on LPS-induced CXCL8 expression. However, we were unsure with the effect of CXCRs on LPS-induced CXCL8 expression. The involvement of CXCRs in CXCL8 expression in our model requires further studies.

CXCL8 promotes tumor progression through regulating construction of tumor microenvironment. It regulates tumor cell proliferation and differentiation, enhancing tumor growth and cell migration. It recruits monocytes that further differentiate into tumor-associated macrophags (TAMs) in the tumor microenvironment. TAMs secrete proinflammatory cytokines and chemokines that enhance tumor inflammation and factors such as VEGF and MMPs that favor angiogenesis. CXCL8 also recruits endothelial progenitor cells that undergo differentiation and proliferation under the regulation of CXCL8 and TAMs. The migrated tumor cells escape into the newly formed blood vessels forming metastasis. Metformin suppresses tumor progression through inhibiting tumor inflammation, tumor cell proliferation and migration, and angiogenesis. We hypothesized that the anticancer properties of metformin are, at least partially, based on inhibition of tumor microenvironment construction through suppressing CXCL8 expression. Our study demonstrated that metformin suppressed LPS-induced CXCL8 expression via inhibiting NF-*κ*B nuclear translocation in HEK293/TLR4 cells. Under the condition we used, metformin dampened cell migration without affecting cell proliferation.
